# The effect of APOE genotype on the delivery of DHA to cerebrospinal fluid in Alzheimer’s disease

**DOI:** 10.1186/s13195-016-0194-x

**Published:** 2016-06-30

**Authors:** Hussein N. Yassine, Varun Rawat, Wendy J. Mack, Joseph F. Quinn, Karin Yurko-Mauro, Eileen Bailey-Hall, Paul S. Aisen, Helena C. Chui, Lon S. Schneider

**Affiliations:** Department of Medicine, Keck School of Medicine of the University of Southern California, Los Angeles, CA USA; Department of Preventive Medicine, Keck School of Medicine of the University of Southern California, Los Angeles, CA USA; Department of Neurology, Oregon Health & Science University, and Portland VA Medical Center , Portland, OR USA; Clinical Research Department, DSM Nutritional Products, Columbia, MD USA; Alzheimer’s Therapeutic Research Institute, Keck School of Medicine of the University of Southern California, Los Angeles, CA USA; Department of Neurology, Keck School of Medicine of the University of Southern California, Los Angeles, CA USA; Department of Psychiatry and the Behavioral Sciences, Keck School of Medicine of the University of Southern California, Los Angeles, USA

**Keywords:** *APOE*, Alzheimer’s disease, Cerebrospinal fluid, Amyloid

## Abstract

**Background:**

Apolipoprotein E (*APOE*) ɛ4 and low cerebrospinal fluid (CSF) amyloid-β42 (Aβ42) levels are predictors for developing Alzheimer’s disease (AD). The results of several studies indicate an interaction between docosahexaenoic acid (DHA) consumption and cognitive outcomes by *APOE* genotype. Our objective in the present study was to examine whether *APOE* ɛ4 genotype and low CSF Aβ42 levels were associated with reduced delivery of DHA to CSF in the Alzheimer’s Disease Cooperative Study-sponsored DHA clinical trial.

**Methods:**

Phospholipid DHA was assayed in the plasma of 384 participants and CSF of 70 participants at baseline. Forty-four of the 70 participants completed the 18-month follow-up visit after allocation to placebo (*n* = 15) or DHA (*n* = 29). Plasma and CSF DHA levels, CSF Aβ42, Tau, and phosphorylated Tau were measured at baseline and after the 18-month intervention. Participants were divided into tertiles based on baseline Aβ42 CSF levels. To assess DHA delivery across the blood-brain barrier, the ratio of CSF to plasma DHA levels was calculated.

**Results:**

At baseline, there were no significant differences between CSF or plasma phospholipid DHA levels by CSF Aβ42 tertiles or ɛ4 status. After 18 months of DHA supplementation, participants at the lowest Aβ42 tertile had significantly lower CSF DHA levels (*p* = 0.01) and lower CSF-to-plasma DHA ratios (*p* = 0.05) compared to the other tertiles. Baseline CSF Aβ42 levels were significantly lower in ɛ4 carriers than in ɛ4 noncarriers (*p* = 0.01). Participants carrying the ɛ4 allele (*n* = 25) demonstrated a less pronounced increase in CSF DHA level compared with noncarriers (*n* = 4), with a possible interaction effect between treatment and APOE genotype (*p* = 0.07).

**Conclusions:**

APOE ɛ4 allele and lower CSF Aβ42 levels were associated with less transport of DHA to CSF. Brain amyloid pathology may limit the delivery of DHA to the brain in AD.

**Trial Registration:**

Clinicaltrials.gov identifier: NCT00440050. Registered on 22 Feb 2007.

## Background

Among the genes associated with late-onset Alzheimer’s disease (AD), the gene encoding for apolipoprotein E (*APOE*) ɛ4 has the strongest correlation with disease onset [1–3]. The ɛ4 isoform is expressed in about 15 % of the general population. However, it is present in about 40 % of patients with AD. Individuals with one ɛ4 allele have a 3- to 4-fold increased propensity toward developing AD, which increases to 12-fold for individuals homozygous for the ɛ4 allele [2]. Furthermore, *APOE* ɛ4 has a similar effect on age of AD onset, with carriers of the ɛ4 allele developing AD symptoms earlier than the ɛ3 carriers. Conversely, individuals with the ɛ2 allele have a lower risk of developing AD [3].

Docosahexaenoic acid (DHA), an omega-3 polyunsaturated fatty acid (n-3 PUFA) is important for brain health, as humans may not produce enough of it de novo. DHA is required for maintenance of neuronal membranes, amyloid-β (Aβ) clearance, and modulation of inflammation [4]. DHA is involved in a variety of physiological processes, including aging, memory formation, synaptic membrane function, photoreceptor biogenesis and function, and neuroprotection. The levels of plasma DHA correlate with brain DHA content [5], and they are reduced in AD brains [6–8]. Despite a large number of observational studies linking DHA intake to cognitive health [4], randomized controlled trials in which investigators tested the effect of DHA intake on cognitive function presented conflicting results [9–13]. Several studies suggested that carriers and non-carriers of *APOE* ɛ4 respond differently to DHA supplementation [12, 14–18], with little or no effect of DHA supplementation [12] and no relationship between the omega-3 on erythrocyte membranes with measures of cognition [17, 18] in ɛ4 carriers with cognitive impairment.

DHA crosses the blood-brain barrier after supplementation, but little is known about the factors that regulate its delivery to the brain [19]. A preclinical study in human APOE replacement mice demonstrated reduced delivery of ^14^C -labeled DHA to the brain in the ɛ4 compared with the ɛ3 and ɛ2 human replacement mice [20]. In a different study, Calon et al. measured cerebral uptake of ^14^C-DHA in 3xTg-AD mice that are prone to brain amyloid deposition. Those investigators found a 25 % (*p* < 0.001) decrease of brain transport coefficients of ^14^C-DHA in this model of AD compared with nontransgenic littermates [21]. To our knowledge, researchers examined the effect of DHA supplementation on cerebrospinal fluid (CSF) levels in humans in only two studies. [12, 22]. In one study by Freund-Levi et al., CSF DHA levels were increased after 6 months supplementation with 2.3 g/day of a combination of n-3 PUFA, but the effect of *APOE* genotype on CSF delivery of DHA was not assessed. In the other study, a randomized, placebo-controlled clinical trial sponsored by the Alzheimer’s Disease Cooperative Study (ADCS), researchers tested the effect of 2 g/day of DHA supplementation on cognitive function in AD over the course of 18 months, and reported a significant increase in CSF DHA after supplementation [12]. The primary study outcomes were the Alzheimer’s Disease Assessment Scale-Cognition (ADAS-cog) and Clinical Dementia Rating. Both cognitive scores did not improve after DHA supplementation [12]. A preplanned secondary analysis of the ADCS-sponsored trial demonstrated cognitive improvements in both ADAS-cog and the Mini Mental State Examination (MMSE) in non-carriers of the *APOE* ɛ4 allele. We hypothesized that carrying the *APOE* ɛ4 allele and cerebral amyloidosis as indexed by lower CSF Aβ42 levels limit the delivery of DHA to the brain. Therefore, we assessed the amount of DHA in CSF after the intervention by *APOE* ɛ4 genotype and baseline CSF Aβ42 levels in the placebo and treatment arms in this ADCS-sponsored DHA trial.

## Methods

### Overview

The data were obtained from a completed, randomized, double-blind, placebo-controlled trial that was sponsored by the ADCS, a consortium of academic medical centers and private AD clinics funded by the National Institute on Aging. Fifty-one U.S. centers participated in this trial after obtaining approval from their local institutional review boards.

Individuals with probable AD were eligible if (1) their MMSE score was between 14 and 26, (2) they were medically stable, (3) they consumed on average no more than 200 mg/day of DHA (as assessed by a brief 7-item food frequency questionnaire [23]), and (4) they were not taking DHA or omega-3 fatty-acid supplements. A total of 384 of 402 study trial participants provided plasma for DHA measurements. Participants were randomly allocated to placebo or 2 g of DHA (supplied by DSM Nutritional Products, Columbia, MD, USA) and observed for 18 months. A total of 295 participants completed the trial while taking study medication (DHA group 171, placebo group 124). All participants without contraindications to CSF examination (e.g., anticoagulation) were invited to participate in the CSF study. In these individuals, lumbar puncture was performed the morning after an overnight fast at baseline and 18 months following randomization.

The study drug was an algae-derived DHA (DHASCO oil) obtained from DSM Nutritional Products, administered as four capsules, dosed as 1 g twice per day for a total daily dose of 2 g/day of DHA. DHASCO oil contains approximately 45–55 % DHA by weight and does not contain eicosapentaenoic acid (EPA). The DHA dose was selected on the basis of evidence that plasma levels increase in a dose-dependent manner up to approximately 2 g/day, while at higher DHA doses no further increase in plasma DHA is observed [24]. Placebo (corn/soy oil) capsules were identical in appearance.

In the fatty-acid analysis, plasma phospholipid fatty-acid levels were determined at DSM Nutritional Products using established methods [24] with modifications for CSF analysis. Briefly, plasma total lipids were extracted from 400 μl of plasma using the methods of Folch et al. [25]. The plasma phospholipids were isolated by thin-layer chromatography using 60/40/3 vol/vol/vol hexane/ether/acetic acid on 20 × 20 silica gel 60 plates with 250-μm thickness. CSF total lipids were extracted from 4 ml of CSF, also using the methods of Folch et al. Tricosanoic free fatty acid (23:0) was added to each sample as an internal standard. The plasma phospholipids and CSF total lipids were saponified with 0.5 N methanolic sodium hydroxide, and the fatty acids were converted to methyl esters with 14 % boron trifluoride/methanol at 100 °C for 30 minutes [26]. Fatty-acid methyl esters were analyzed by gas-liquid chromatography using a Hewlett Packard 6890 chromatograph (Agilent Technologies, Santa Clara, CA, USA) equipped with a flame ionization detector. The fatty-acid methyl esters were separated on a 30-m FAMEWAX capillary column (0.25-mm diameter, 0.25-μm coating thickness; Restek, Bellefonte, PA, USA) using hydrogen at a flow rate of 2.1 ml/minute. The chromatographic run parameters included an oven starting temperature of 130 °C that was increased at a rate of 6 °C/minute to 225 °C, where it was held for 20 minutes before being increased to 250 °C at a rate of 15 °C/minute, with a final hold of 5 minutes. The injector and detector temperatures were constant at 220 °C and 230 °C, respectively. Plasma phospholipids were run at a 48:1 split flow, and the CSF total lipids were run at a 20:1 split flow. Peaks were identified by comparison of retention times with external fatty-acid methyl ester standard mixtures obtained from Nu-Chek Prep (Elysian, MN, USA). The fatty-acid profiles were expressed as a percentage of the total fatty acid in micrograms (weight percent). *APOE* genotype was assessed in the research laboratory. Aβ42 and Tau in the CSF were measured by using a dual-antibody sandwich enzyme-linked immunosorbent assay [27] at the University of Pennsylvania Research Lab.

### Statistical analysis

Mean (SD) or median (25th–75th percentile range) values for non-normally distributed data were computed. The study group was divided into tertiles based on CSF Aβ42 levels at baseline. The ratio of CSF to plasma DHA was calculated as an index of DHA transport across the blood-brain barrier. The dependent variables were (1)18 months CSF and plasma phospholipid DHA levels, (2) ratio of CSF to plasma DHA levels at 18 months, and (3) the difference in DHA levels in CSF and plasma phospholipid at baseline and 18 months after supplementation. The independent variables were Aβ group (tertiles), treatment group, *APOE* genotype, and the interaction between the treatment group and *APOE* genotype using a linear regression model. Pearson or Spearman correlation tests were used to correlate the variables. Baseline plasma phospholipid and CSF DHA levels by *APOE* groups were explained by a linear regression model. Linear modeling was also used to explain the relationship of baseline measures of CSF Aβ42 (independent variable) with the 18-month change in CSF DHA (dependent variable); this analysis was adjusted for baseline CSF DHA and *APOE* genotype. Significance was defined as *p* < 0.05. The data were analyzed using the program R version 3.2.3.

## Results

**Fig. 1:**
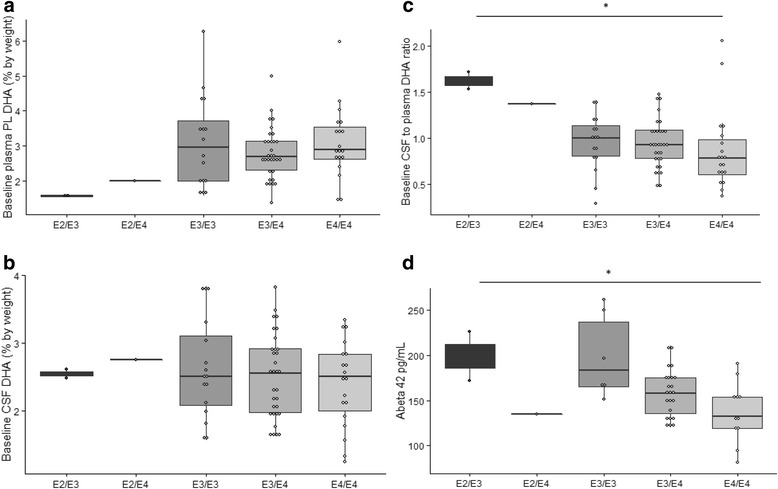
Baseline CSF DHA levels by *APOE* status. The distributions of baseline plasma phospholipid and CSF DHA, as well as the ratio of CSF to plasma DHA, are demonstrated by *APOE* genotype (*n* = 70). There were no significant differences in (**a**) plasma phospholipid or (**b**) CSF DHA levels by *APOE* genotype. **c** The ratio of CSF to plasma DHA by *APOE* genotype at baseline significantly differed between the *APOE* genotype groups (*p* = 0.03 for group comparison). **d** CSF Aβ42 levels were significantly lower in ɛ4 carriers at baseline (*p* = 0.01 for group comparison). DHA levels are reported as a percentage by weight. The groups were compared using linear regression with DHA level or CSF-to-plasma DHA ratio as the dependent variable and groups as the covariate.*p < 0.05 for group comparison. *Aβ42* amyloid-β42, *APOE* apolipoprotein E, *CSF* cerebrospinal fluid, *DHA* docosahexaenoic acid, *PL Phospholipids*

Plasma (*n* = 384) and CSF samples (*n* = 70) from participants in this ADCS trial were assayed for DHA levels at the baseline visit. The 70 participants who consented to lumbar puncture included carriers of ɛ2/ɛ3 (*n* = 2), ɛ2/ɛ4 (*n* = 1), ɛ3/ɛ3 (*n* = 16), ɛ3/ɛ4 (*n* = 32), and ɛ4/ɛ4 (*n* = 19). Forty-four of the 70 CSF substudy participants completed the second lumbar puncture at the 18-month visit after allocation to either the placebo (*n* = 15) or DHA (*n* = 29) treatment group. Among those allocated to DHA were carriers of ɛ3/ɛ3 (*n* = 4), ɛ3/ɛ4 (*n* = 17), and ɛ4/ɛ4 (*n* = 7), and one participant carried the ɛ2/ɛ4 allele. Baseline levels of plasma phospholipid DHA (*n* = 384, *p* = 0.61) and total CSF DHA (*n* = 70, *p* = 0.44) did not differ between *APOE* ɛ4 carriers and noncarriers. The distribution of baseline CSF DHA levels and plasma phospholipid DHA levels, as well as the ratio of CSF to plasma DHA, in all 70 participants is shown in Fig. 1. These findings indicated that plasma phospholipid DHA (*p* = 0.8) (Fig. 1a) and CSF DHA (*p* = 0.7) (Fig. 1b) did not differ by *APOE* genotype at baseline. The ratio of CSF to plasma DHA was significantly different among the *APOE* genotype groups (*p* = 0.03 for groupwise comparison) (Fig. 1c), with the largest differences apparent between ɛ2 carriers and ɛ4 homozygotes. However, the significance of this finding is limited by the small number of ɛ2 carriers in this group (*n* = 3). Baseline CSF and plasma DHA levels were significantly correlated (*r* = 0.3, *p* = 0.01); this correlation did not differ by ɛ4 status. Of the 70 participants, 44 had measurements of CSF Aβ42. *APOE* ɛ4 carriers had lower CSF Aβ42 levels compared with non-carriers (*p* = 0.01) (Fig. 1d).

**Fig. 2:**
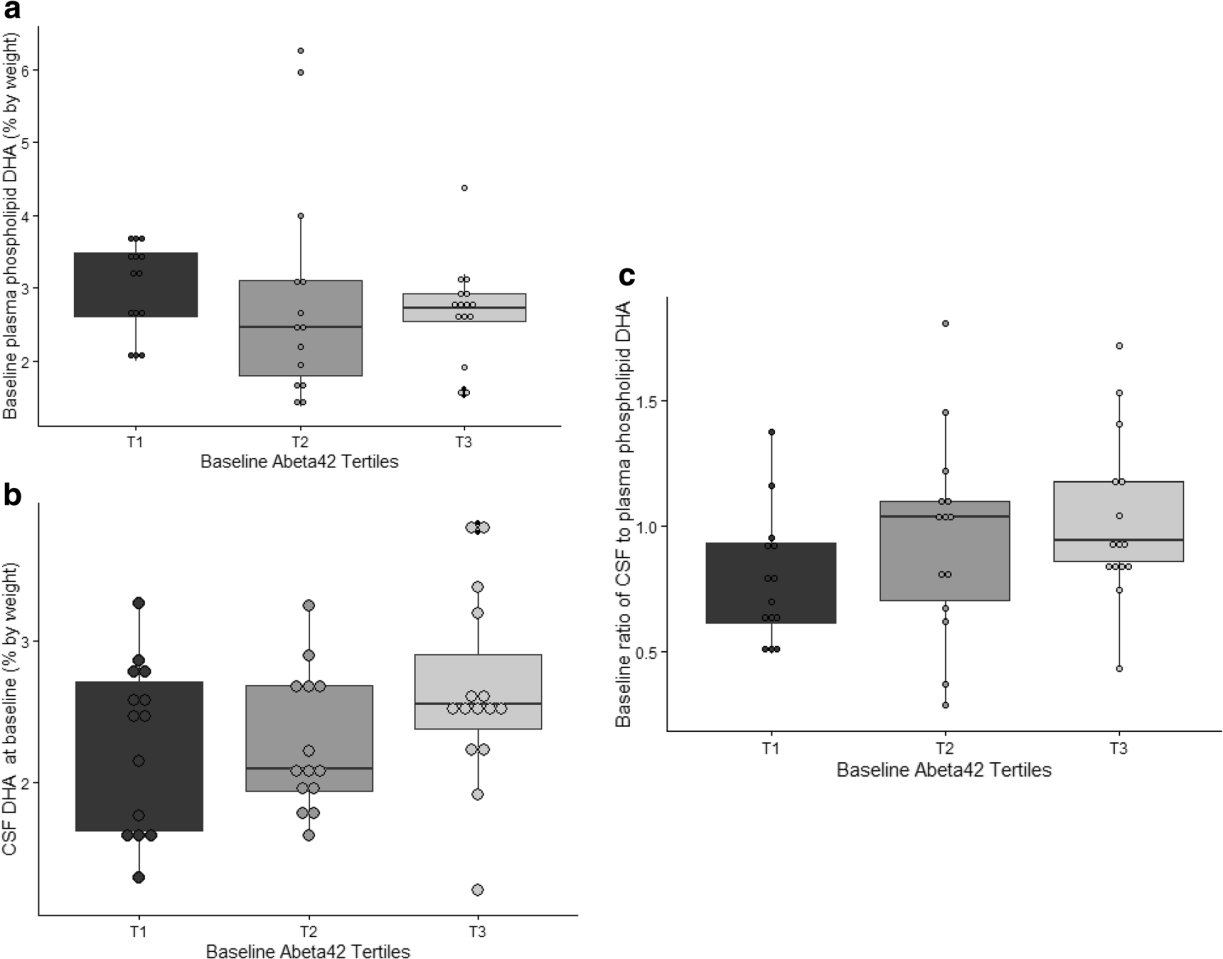
Baseline plasma phospholipid and CSF DHA levels by CSF Aβ42 tertiles. Baseline phospholipid and CSF DHA measurements were compared in three groups of participants based on CSF Aβ42 tertiles at baseline (*n* = 44, T1 = Aβ42 levels <147 pg/ml, T2 = Aβ42 levels between 147 and 174 pg/ml, and T3 = Aβ42 levels >174 pg/ml). The ratio of CSF to plasma DHA was calculated as an index of DHA transport across the blood-brain barrier (**a** and **b**). There was no significant difference in plasma phospholipid of CSF DHA by Aβ42 tertiles at baseline. **c** A trend was observed for lower baseline CSF to plasma DHA (*p* = 0.15 for three-way group comparison, *p* = 0.19 for difference between T1 and T2, and *p* = 0.06 for difference between T1 and T3) in the lowest tertile of Aβ42. *Aβ42* amyloid-β42, *CSF* cerebrospinal fluid, *DHA* docosahexaenoic acid

To understand the effect of baseline CSF Aβ42 levels on DHA levels, the study group was divided into CSF Aβ42 tertiles (T1 = Aβ42 levels <147 pg/ml, T2 = Aβ42 levels between 147 and 174 pg/ml, T3 = Aβ42 levels >174 pg/ml). At baseline, plasma phospholipid or CSF DHA did not differ between the groups (Fig. 2a and b). The participants at the lowest tertile of CSF Aβ42 had lower mean CSF-to-plasma DHA ratios than the other two groups; however, this difference did not reach statistical significance (*p* = 0.15 for three-way group comparison, *p* = 0.19 for difference between T1 and T2, and *p* = 0.06 for difference between T1 and T3) (Fig. 2c).

Plasma phospholipid DHA was assessed in 195 DHA-treated participants at baseline and 18 months following DHA supplementation. Among the 195, 119 carried the ɛ4 allele and 76 were ɛ4 noncarriers. A 300 % increase in plasma phospholipid DHA was observed (from 3.18 weight percentage at baseline to 9.82 weight percentage at 18 months; *p* < 0.001). CSF DHA was assessed in 44 participants at baseline and 18 months. The increase in plasma DHA level was greater than the increase in CSF DHA. A 38 % increase in DHA CSF levels was observed in participants in the DHA treatment group (2.53 weight percentage at baseline and 3.46 weight percentage at 18 months; *p* < 0.001). In participants allocated to DHA (*n* = 29), the change in DHA levels from baseline to 18 months in CSF significantly correlated with the 18-month change in plasma (*r* = 0.61, *p* < 0.001).

**Fig. 3:**
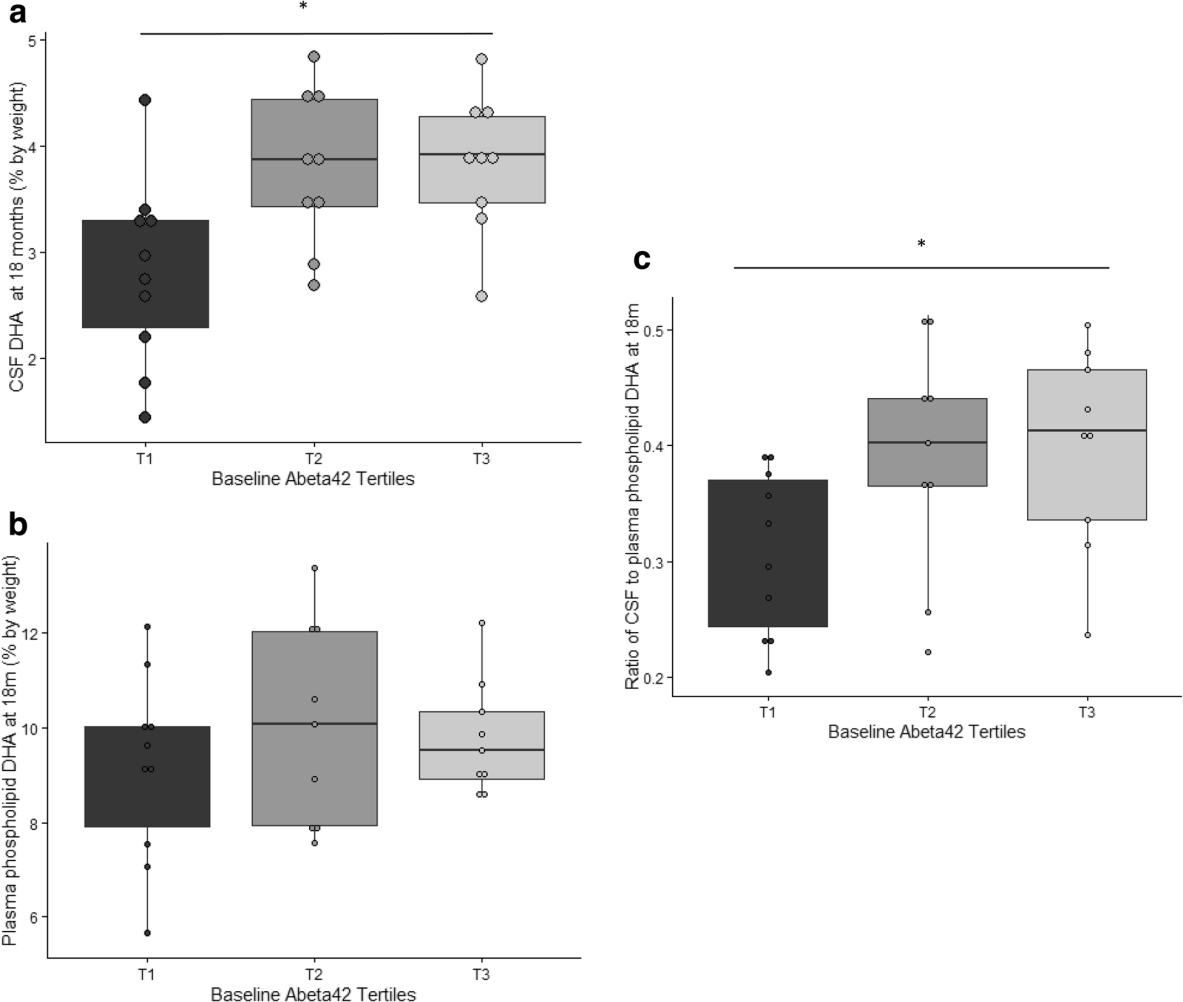
Plasma phospholipid and CSF DHA at 18 months. The distribution of phospholipid and CSF DHA measurements at 18 months by baseline CSF Aβ42 tertiles in participants allocated to DHA (*n* = 29) is illustrated. **a** There was no significant difference in plasma phospholipid DHA by Aβ42 tertiles after 18 months of DHA. **b** and **c** After 18 months of DHA supplementation, there was a significant decrease in CSF DHA levels in participants in the the first Aβ42 tertile (*p* = 0.01 for three-way group comparison, *p* = 0.01 for difference between T1 and T2, and *p* = 0.007 for difference between T1 and T3) and a significantly lower increase in the CSF-to-plasma DHA ratio (*p* = 0.05 for three-way group comparison, *p* = 0.05 for difference between T1 and T2, and *p* = 0.03 for difference between T1 and T3). The groups were compared using a linear regression model. *p < 0.05 for group comparison. *Aβ42* amyloid-β42, *CSF* cerebrospinal fluid, *DHA* docosahexaenoic acid

**Fig. 4:**
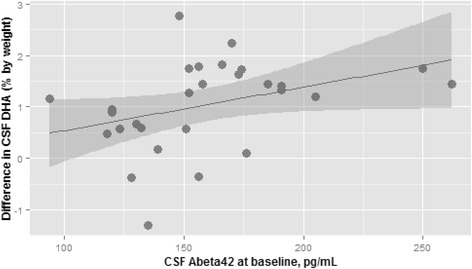
Association of CSF Aβ42 with the change in CSF DHA. Baseline CSF Aβ42 was significantly associated with the change in CSF DHA after supplementation (*r* = 0.37, *p* = 0.05). The change in DHA was calculated as the difference between 18-month CSF DHA levels and the levels at baseline. The correlation was obtained using Spearman’s correlation test. *Aβ42* amyloid-β42, *CSF* cerebrospinal fluid, *DHA* docosahexaenoic acid

Participants at the lowest tertile of Aβ42 had significantly lower mean CSF DHA levels after supplementation (*p* = 0.01 for three-way group comparison, *p* = 0.01 for difference between T1 and T2, and *p* = 0.007 for difference between T1 and T3) (Fig. 3a). The differences in CSF DHA levels among the Aβ42 groups at 18 months persisted after adjusting for *APOE* genotype (*p* = 0.03 for three-way group comparison). In contrast, there was no significant difference in plasma DHA levels after supplementation by Aβ42 tertiles (Fig. 3b). The ratio of CSF to plasma DHA ratio was significantly lower after 18 months of DHA supplementation in participants at the lowest tertile of Aβ42 (*p* = 0.054 for three-way group comparison, *p* = 0.05 for difference between T1 and T2, and *p* = 0.03 for difference between T1 and T3) (Fig. 3c). When CSF Aβ42 was analyzed as a continuous variable, CSF Aβ42 levels at baseline were associated with the 18-month change in CSF DHA (*r* = 0.37, *p* = 0.05) (Fig. 4). This relationship remained significant after we adjusted for baseline DHA levels (*p* = 0.037) but was attenuated after we excluded the two CSF Aβ42 values greater than 250 pg/ml (*r* = 0.35, *p* = 0.07). After adjusting these models for *APOE* genotype, we found that the relationship of the 18-month change in DHA and baseline Aβ42 levels became less significant (*p* = 0.1).

**Fig. 5:**
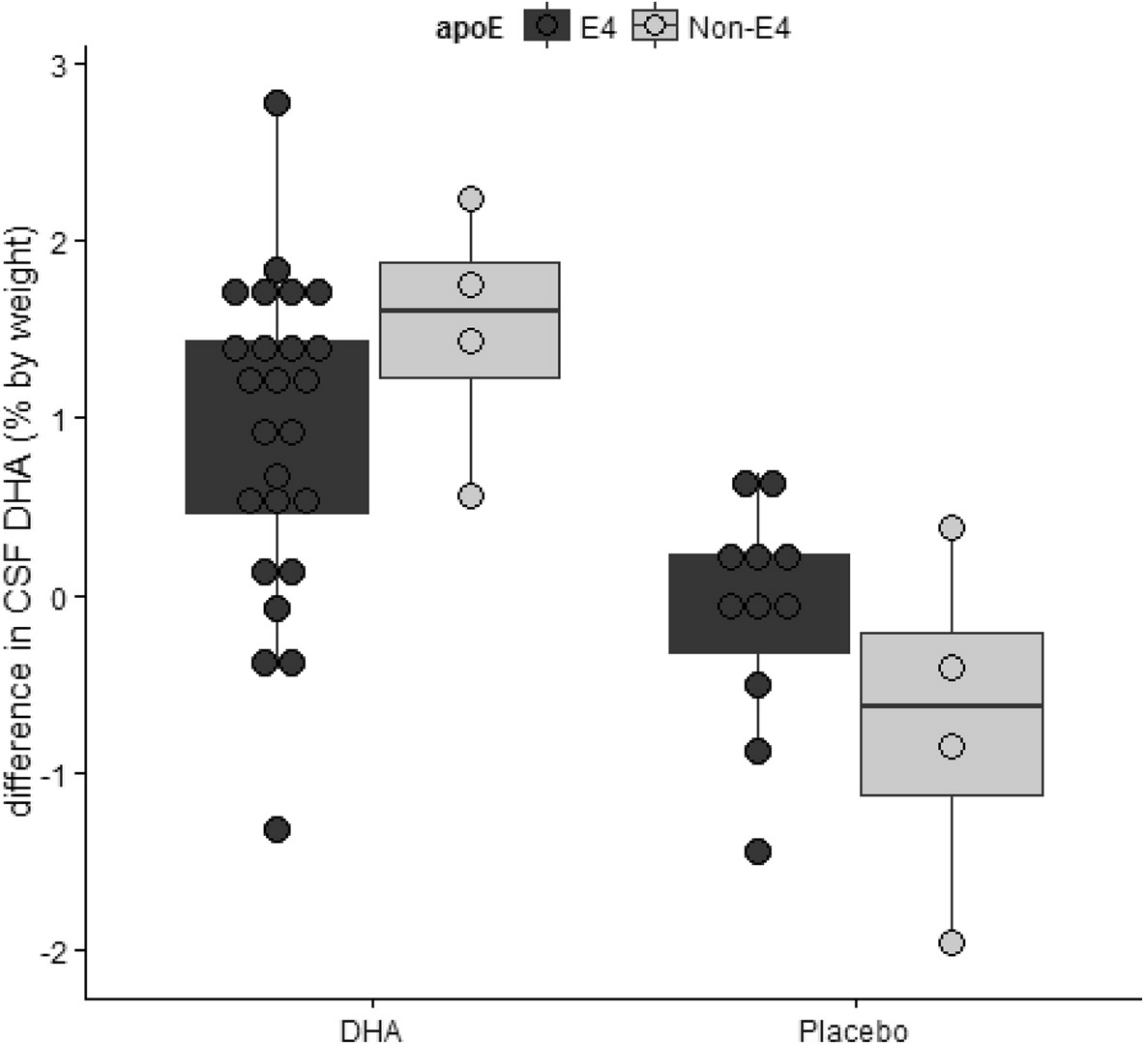
Change in CSF DHA by *APOE* status and treatment arm. The effect of DHA treatment vs. placebo on CSF DHA levels by *APOE* genotype is illustrated. The increases in DHA levels in the CSF were less pronounced in carriers of the ɛ4 allele. All ɛ4 noncarriers had increased CSF DHA levels after allocation to DHA treatment. In contrast, 6 of the 25 ɛ4 carriers did not increase DHA levels after DHA supplementation. There was a suggestion for an interaction effect between *APOE* genotype and treatment arm on CSF DHA levels (*p* = 0.07). The data were modeled using multivariate linear regression with the change in CSF DHA as the dependent variable and *APOE* and treatment arm as the independent variables. *Aβ42* amyloid-β42, *APOE* apolipoprotein E, *CSF* cerebrospinal fluid, *DHA* docosahexaenoic acid

There was a suggestion that *APOE* ɛ4 status modified the DHA effect on the 18-month change in CSF, but not plasma phospholipid DHA levels. The 18 months, the increase in plasma phospholipid DHA did not differ by *APOE* ɛ4 allele (*p* = 0.8). In contrast, a possible interaction between *APOE* genotype and treatment at the 18-month time point in CSF DHA (*p* = 0.07) was observed (Fig. 5). In the DHA treatment group, over 18 months, the ɛ4 noncarriers’ DHA levels increased by 68 %, whereas the ɛ4 carriers’ CSF DHA levels increased by 37 % (Table 1). All four ɛ4 noncarriers showed increased CSF DHA levels after allocation to DHA treatment. In contrast, 6 of 25 ɛ4 carriers did not have increased CSF DHA levels after supplementation. Tau and phosphorylated Tau (p-Tau) levels in the CSF did not differ between carriers and noncarriers of the ɛ4 allele. Allocation to DHA treatment did not alter the decline in CSF Aβ42 or the change in Tau or p-Tau compared with placebo. Additional information on CSF DHA, Aβ42, Tau, and p-Tau by treatment group is summarized in Table 1.

**Table 1:** Eighteen-month changes with docosahexaenoic acid or placebo treatment by apolipoprotein E ε4 status on CSF DHA, Aβ42, Tau and p-Tau levels

	DHA treated, *APOE* ɛ4 non-carriers (*n* = 4)		Placebo treated, *APOE* ɛ4 non-carriers (*n* = 4)		DHA treated, *APOE* ɛ4 carriers (*n* = 25)		Placebo treated, *APOE* ɛ4 carriers (*n* = 11)	
	Baseline	18 months	Baseline	18 months	Baseline	18 months	Baseline	18 months
DHA,^a^ % by weight	2.21 (0.44)	3.71 (0.71)	2.66 (0.81)	1.95 (0.28)	2.46 (0.59)	3.38 (0.90)	2.28 (0.6)	2.18 (0.50)
Aβ42, pg/ml	208 (55)	189 (51)	190 (36)	183 (36)	150 (30)	140 (30)	151 (35)	145 (33)
Tau, pg/ml	120 (110)	121 (125)	118 (42)	129 (35)	112 (53)	108 (136)	133 (94)	126 (88)
p-Tau,^b^ pg/ml	39 (43)	40 (43)	47 (23)	53 (28)	47 (18)	44 (17)	53 (37)	51(33)

*Abbreviations: Aβ42* amyloid-β42, *APOE* apolipoprotein E, *CSF* cerebrospinal fluid, *DHA* docosahexaenoic acid, *p-Tau* phosphorylated Tau

Data are presented as mean (SD). Two-way analysis of variance was used to compare placebo and treatment groups by genotype

^a^ *p* = 0.07 for an interaction between treatment arm and *APOE* genotype

^b^ *p* = 0.04 for the difference in p-Tau by *APOE* groups

DHA treatment changed the percentage of CSF arachidonic acid (AA). We observed a significant decrease in CSF AA in participants assigned to DHA treatment compared with placebo (change in DHA vs. placebo −2.27 vs. −0.64, *p* = 0.002). The 18-month change in AA did not correlate with the 18-month change in CSF Aβ42 or CSF Tau/p-Tau, and it did not differ by *APOE* group. A previous study indicated that DHA supplementation was associated with decreases in AA transport to the brain [22]. Brain AA uptake determined by positron emission tomography is increased in people with AD [28], and AA is a precursor for inflammatory mediators [29]. The change in CSF EPA did not differ between the placebo and treatment arms in the present study (*p* = 0.2). The change in CSF EPA by *APOE* groups at baseline and after DHA supplementation did not reach statistical significance (*p* = 0.13). These findings were expected, as the DHA product we used did not contain EPA.

## Discussion

The main finding of the ADCS-sponsored DHA trial was that the allocation to DHA treatment did not influence the rate of cognitive decline in patients with dementia [12]. A secondary analysis suggested benefit in ADAS-cog scores in noncarriers of the ɛ4 genotype [12]. We hypothesized that the DHA-associated cognitive improvement in ɛ4 noncarriers could be related to greater CSF DHA delivery. Our hypothesis was based on two recent findings in human ɛ4 allele replacement mice [20] and 3xTg-AD transgenic mouse models with brain amyloid deposition [21] demonstrating less delivery of ^14^C labeled DHA across the blood-brain barrier compared with ɛ4 non-carriers or littermate controls, respectively. The results of the present analysis suggest that (1) individuals with lower pretreatment CSF Aβ42 (both ɛ4 carriers and ɛ4 non-carriers) had reduced CSF DHA levels after supplementation, and (2) carriers of the ɛ4 allele had less pronounced increases in CSF DHA levels following DHA treatment compared with ɛ4 non-carriers. Therefore, it is possible that the lack of cognitive effect of DHA in this study was a result of poor brain delivery in participants with brain amyloid pathology and in those carrying the *APOE* ɛ4 allele.

The major limitation of this study was the small number of ɛ4 noncarriers (*n* = 4). However, the differences in CSF DHA response between carriers and noncarriers of the *APOE* ɛ4 allele may also be represented by the lower CSF Aβ42 levels observed in ɛ4 carriers. Taken together, our results support the concept that the diminished response in the *APOE* ɛ4 carriers could be the result of more severe disease with significant brain amyloidosis.

The results of our study suggest an effect of CSF Aβ42 on baseline CSF DHA levels and CSF-to-plasma DHA ratio. We observed 25 % lower CSF-to-plasma DHA ratio between the first and third tertiles of CSF Aβ42. This observation did not reach statistical significance, likely due to the small sample size (Fig. 2c). After the DHA intervention, however, these differences in CSF DHA levels by CSF Aβ42 groups were significant. The advantage of the DHA intervention is that it reduces variation resulting from potential confounders with baseline measurements (such as differences in seafood consumption or supplement use before the study) and provides a uniform dose of DHA supplementation using a controlled study design. The lower CSF-to-plasma DHA ratio in participants with lower CSF Aβ42 levels suggests a defect in DHA transport to the brain in AD.

One potential mechanism for these findings includes activation of phospholipase A_2_ (PLA_2_). Upon entry into the brain, DHA is trapped by long-chain fatty-acid coenzyme A synthase activity, thereby facilitating its targeting to specific lipid pools, where it is esterified to phospholipid membranes [30]. In the adult brain, DHA is no longer accreted (*accretion* refers to accumulation of DHA during development [31]), and plasma DHA replaces brain consumption [32]. DHA is esterified to phospholipids at the sn-2 position and deesterified by PLA_2_. Brain DHA is highly conserved, but at an energy cost [33]. Upon release by PLA_2_ activity, DHA is immediately reesterified into brain phospholipids. A decrease in both nonesterified DHA and total DHA in the CSF is observed in mild cognitive impairment and AD, as compared with cognitively healthy participants, and is associated with increased PLA_2_ activity [34]. PLA_2_ is a complex family of phospholipases that include calcium-independent phospholipase A_2_ and calcium-dependent phospholipase A_2_ (cPLA_2_). cPLA_2_ can target DHA, AA, and other lipids (such as plasmalogens). Several lines of evidence suggest that calcium-dependent signaling pathways are dysregulated in the neurons of hAPP (amyloidosis-prone) mice, particularly in the hippocampus [35]. We hypothesize that amyloid pathology induces the activity of cPLA_2_ [36, 37] in AD brain regions, reducing brain DHA consumption through liberation of free DHA from CSF and brain phospholipids [38]. These changes have significant implications for AD pathology, as DHA is critical in hippocampal neuronal development and synaptic function [39].

The effect of *APOE* genotype on peripheral DHA metabolism is not clear. One study demonstrated a less pronounced increase in DHA associated with triglyceride or cholesterol ester after 6 weeks of DHA + EPA supplementation in ε4 carriers vs. noncarriers [40]. However, changes in DHA associated with triglyceride-rich particles by *APOE* genotype were not observed in the SATgenε study [41]. Our results did not reveal that plasma phospholipid DHA levels differed by *APOE* ɛ4 status at baseline or following DHA supplementation. In a population study with a larger sample size (*n* = 1135), however, greater fish consumption was associated with greater increases in plasma phospholipid DHA only in ɛ4 noncarriers [42].

There is evidence in several studies other than the ADCS-sponsored DHA trial [12] that the *APOE* ɛ4 allele can modulate the response to DHA supplementation. In an Alzheimer’s Disease Neuroimaging Initiative study that included cognitively healthy persons, patients with mild cognitive impairment, and patients with AD, the association of fish oil with ADAS-cog and brain volume was observed only in ɛ4-negative patients [16]. In 2005, Huang et al. [17] examined fish oil use in the Cardiovascular Health Cognition Study (*n* = 2233) among participants who developed dementia after several years of follow-up. Their study demonstrated that *APOE* status was an important determinant in modulating the effect of n-3 intake on cognitive status, with only ɛ4 non-carriers being responsive to n-3 intake. In another longitudinal study, Whalley et al. reported a stronger association of red blood cell n-3 index with cognitive scores in ɛ4 non-carriers compared with carriers [18]. In contrast, three studies of participants without AD (Bordeaux sample of the three-city study [1999–2006, *n* = 1228] [43], Chicago Health and Aging Project [1993–2000, *n* = 818] [44], and the Memory and Aging Project clinical neuropathological cohort study [2004–2013, *n* = 286] [45]) suggest cognitive benefit in ε4 carriers with greater n-3 levels, or less brain AD neuropathology with weekly seafood consumption.

In summary, these studies indicate an interaction between ɛ4 allele and DHA efficacy, with the ɛ4 allele potentially limiting the effectiveness of DHA supplementation on cognition later in the disease process. It would be important to assess the delivery of DHA to the brain by *APOE* ε4 status before the onset of detectable brain amyloidosis and AD. These studies would then clarify whether cognitively healthy younger *APOE* ε4 carriers have a chronic defect in DHA brain delivery years before the onset of neurodegeneration, presenting with an opportunity for DHA supplementation to prevent or slow the progression of AD.

## Conclusions

To our knowledge, this is the first study to demonstrate changes in CSF DHA levels in relation to *APOE* genotypes and CSF Aβ42 peptide levels. Our main finding suggests decreased DHA delivery to CSF in participants with lower CSF Aβ42 peptide and in carriers of the *APOE* ε4 allele. These findings can help explain the lack of efficacy of DHA in participants with established AD. Future studies are needed to clarify if differences in DHA transport in participants carrying the *APOE* ε4 allele appear earlier in life, before the onset of cognitive decline.

## Abbreviations

AA, arachidonic acid; Aβ42, amyloid-β42; AD, Alzheimer’s disease; ADAS-cog, Alzheimer’s Disease Assessment Scale-Cognition; ADCS, Alzheimer’s Disease Cooperative Study; *APOE*, apolipoprotein E; cPLA_2_, calcium-dependent phospholipase A_2_; CSF, cerebrospinal fluid; DHA, docosahexaenoic acid; EPA, eicosapentaenoic acid; MMSE, Mini Mental State Examination; n-3 PUFA, ω-3 polyunsaturated fatty acid; PLA_2_, phospholipase A_2_; p-Tau, phosphorylated Tau; Tg, transgenic
